# Perpendicular ion heating in turbulence and reconnection: magnetic moment breaking by coherent fluctuations

**DOI:** 10.1017/S0022377825101177

**Published:** 2026-02-23

**Authors:** Alfred Mallet, Kristopher Klein, Benjamin Divakar Giles Chandran, Tamar Ervin, Trevor A. Bowen

**Affiliations:** 1 Space Sciences Laboratory, University of California, Berkeley, CA 94720, USA; 2 Lunar and Planetary Laboratory, University of Arizona, Tucson, AZ 85719, USA; 3 Space Science Center and Department of Physics, University of New Hampshire, Durham, NH 03824, USA; 4 Department of Physics, University of California, Berkeley, CA 94720, USA

**Keywords:** astrophysical plasmas, plasma heating, space plasma physics

## Abstract

We study the interaction of an ion with a fluctuation in the electromagnetic fields that is localised in both space and time. We study the scale dependence of the interaction in both space and time, deriving a generic form for the ion’s energy change, which involves an exponential cutoff based on the characteristic time scale of the electromagnetic fluctuation. This leads to diffusion in energy in both 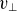 and 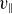 . We show how to apply our results to general plasma physics phenomena, and specifically to Alfvénic turbulence and to reconnection. Our theory can be viewed as a unification of previous models of stochastic ion heating, cyclotron heating and reconnection heating in a single theoretical framework.

## 1. Introduction

In both the solar corona and solar wind, observations show that proton heating is typically much greater than the electron heating, with minor ions heated even more strongly, and moreover that ion heating is mainly perpendicular to the magnetic field (Kohl *et al*. 1998; Marsch *et al*. 1982, 2004; Antonucci, Dodero & Giordano 2000; Hellinger *et al*. 2006; Kasper *et al*. 2017; Bowen *et al*. 2020). Characterising ion heating is therefore essential for the thermodynamics of this system (Parker 1965). More generally, correctly parametrising the ratio of ion-to-electron heating in plasma turbulence is of great interest for the interpretation of many remote astrophysical observations (Chael *et al*. 2018).

What is the source of free energy for the observed heating? One successful model is heating from the Alfvénic plasma turbulence ubiquitous in the solar wind (Belcher & Davis 1971; Chen 2016; Chen *et al*. 2020) and corona (De Pontieu *et al*. 2007); the fluctuation amplitudes are consistent with the observed plasma heating (Chandran & Hollweg 2009; Cranmer *et al*. 2009), suggesting that the solar wind is accelerated and locally heated by the dissipation of these turbulent fluctuations. It is worth noting that because the turbulence has only a very small compressive component (Klein *et al*. 2012), the ion heating within the gyrokinetic approximation would be much smaller to explain the observations (Schekochihin *et al*. 2009, 2019; Kawazura *et al*. 2020).

Several theoretical models have been proposed to explain ion heating in turbulence. First, cyclotron resonant heating (Hollweg & Isenberg 2002; Chandran *et al*. 2010; Isenberg & Vasquez 2011, 2019; Bowen *et al*. 2024) occurs when, in the frame moving with an ion’s parallel velocity 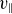 , a wave’s frequency 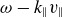 matches the gyrofrequency 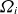 : hence, ‘resonant’, 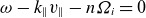 . This is often discussed in the framework of quasilinear theory (Kennel & Engelmann 1966; Stix 1992), where the resulting diffusion of energy in phase space is derived assuming a spectrum of infinite plane waves and considering only the resonant response.

Another important model, closer in approach to that of the present paper, is stochastic heating (McChesney, Stern & Bellan 1987; Chandran *et al*. 2010), in which ions random-walk in energy due to uncorrelated kicks from ion-scale fluctuations. In the model of Chandran *et al*. (2010), this results in a heating rate 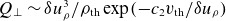 , where 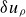 is the amplitude of 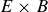 velocity fluctuations at the gyroscale 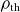 , and the exponential suppression factor was added empirically to account for the near-conservation of the magnetic moment at low frequencies and small amplitudes. One advantage of stochastic heating as opposed to cyclotron resonant heating is that it does not require an exact resonance nor does it assume that the fluctuations resemble infinite plane waves. This allows one to easily incorporate the observed intermittent probability distribution of fluctuation amplitudes (Chandran, Schekochihin & Mallet 2015; Mallet & Schekochihin 2017) at the gyroscale, which can dramatically increase the predicted heating rate (Mallet *et al*. 2019; Cerri, Arzamasskiy & Kunz 2021).

Observations (Chen *et al*. 2011) show that the solar wind turbulence is highly anisotropic: fluctuations have very different characteristic length scales parallel ( 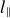 ) and perpendicular ( 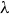 ) to the background magnetic field, 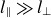 . Modern turbulence theories (Goldreich & Sridhar 1995; Boldyrev 2006) explain this in terms of a critical balance between characteristic time scales associated with linear propagation ( 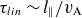 , with 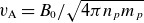 the Alfvén velocity based on the mean magnetic field 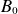 ) and nonlinear interactions ( 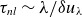 ): the cascade time 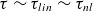 , whence 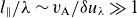 . Since the fluctuating field amplitude 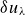 at scale 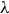 is an increasing function of 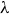 , at progressively smaller scales, the anisotropy 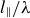 increases. This means that the fluctuations remain relatively low frequency, with 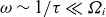 , where 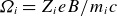 is the ion gyrofrequency. This poses a challenge: the magnetic moment 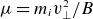 is conserved to all orders in 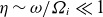 (Kruskal 1962), and so the usual perturbation theory would suggest that perpendicular ion heating should be irrelevant for such anisotropic, small-amplitude turbulence, in contrast to the observations. It is worth noting that ‘to all orders’ is not the same as ‘exactly’: as an example (that will be important in this paper), 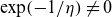 , but is ‘zero to all orders’ if 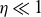 , since all derivatives vanish as 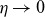 .

In addition to turbulence, magnetic reconnection has been proposed as a mechanism for coronal heating (Klimchuk 2015) and also as a heating mechanism within the turbulence itself (Shay *et al*. 2018). In fact, turbulent heating and reconnection heating may not be as distinct as traditionally thought. The turbulent cascade naturally leads to the formation of extended current sheets (Boldyrev 2006; Chandran *et al*. 2015; Mallet & Schekochihin 2017), which reconnect once their width becomes sufficiently small (Boldyrev & Loureiro 2017; Cerri & Califano 2017; Comisso *et al*. 2017; Franci *et al*. 2017; Loureiro & Boldyrev 2017*a*; Mallet, Schekochihin & Chandran 2017; Vech *et al*. 2018; Dong *et al*. 2022). Similarly, approaching the problem from the ‘reconnection end’, extended reconnecting current sheets are often violently unstable, leading naturally to strong turbulence (Loureiro, Schekochihin & Cowley 2007; Bhattacharjee *et al*. 2009; Huang & Bhattacharjee 2016). Seeking to explain preferential heavy-ion heating in the corona and solar flares, Drake *et al*. (2009*a*) developed a theory of perpendicular ion heating in reconnection exhausts, supported by numerical simulations. Drake *et al*. (2009*b*) showed that in guide-field reconnection, strong ion heating only occurs if the characteristic time scale to transit the exhaust is shorter than the ion’s gyroperiod. This behaviour has similarities to the stochastic heating in turbulence.

All three of cyclotron-resonant, stochastic and reconnection perpendicular ion heating share a common feature: they require the conservation of the magnetic moment to be broken. In the case of stochastic and reconnection heating, this leads to a ‘threshold’ which must be satisfied for strong ion heating to be possible. Likewise, in cyclotron resonant heating, the resonance condition must be satisfied (we will argue that this ‘sharper’ behaviour is a consequence of the plane-wave assumption). Johnston, Squire & Meyrand (2025) noticed the similarities between cyclotron-resonant and stochastic heating, and found that the heating in their test-particle simulations was well described by a single exponential suppression factor modelling both cyclotron-resonant heating in imbalanced turbulence and stochastic heating in balanced turbulence. In related work in a different physical setting, namely electron scattering in the radiation belts, a similarly close relationship between electron scattering by strongly nonlinear coherent structures (electron holes) and quasilinear theory was also previously discussed by Vasko *et al*. (2017; 2018).

In this paper, we develop a new framework that describes perpendicular ion heating. We analytically study the response of an ion to a localised, coherent fluctuation in the electromagnetic fields, with the fluctuating electric 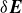 and magnetic 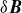 fields tending to zero at 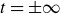 , an approach to our knowledge first taken by Krall & Rosenbluth (1964) for electric field fluctuations and for general adiabatic invariants by Landau & Lifshitz (1976). Quite generically, we find that the perpendicular ion kinetic energy 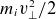 changes by an amount of order
(1.1)

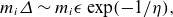

where 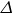 is the change in 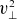 , 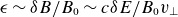 is the normalised amplitude of the fluctuations and 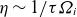 , where 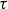 is a characteristic time scale over which 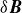 and 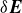 vary. The threshold for strong ion heating to occur is encoded in the exponential factor: 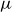 -conservation is lost when 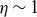 and the fluctuations vary significantly over one gyroperiod. For 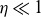 , the magnetic moment is conserved to all orders, but not exactly: for many systems, this is enough to provide significant heating over long time scales. After setting up the system of equations (§ 2), in § 3, we proceed to expand the amplitude of the fluctuating fields, deriving analytic expressions for the change in perpendicular and parallel energy as well as how this depends on the length scale of the fluctuations. We also derive general formulae for the diffusion coefficient and heating rate, and outline how our theory should be applied to different physical systems. We then explicitly show how our results apply to both Alfvénic turbulence (§ 4) and to reconnection (§ 5). Finally, we discuss the relationship of our model to earlier theories, and what the implications of our results are for astrophysical and space plasma turbulence and reconnection heating.

## 2. Normalised equations

The equations of motion for an ion of charge 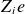 and mass 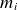 in a general electromagnetic field are
(2.1)

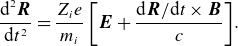




For the magnetic and electric field, we take
(2.2)

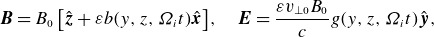

where we assume 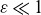 , and 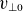 is the perpendicular ion velocity at 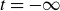 . Our neglect of 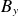 and 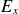 will not change the physical conclusions of our calculation (while making it somewhat less cumbersome), but ignoring 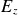 and fluctuations in 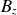 removes the possibility of Landau and transit-time energisation of the particle: we wish to focus solely on the cyclotron interaction. If this makes one uncomfortable, it may be justified by considering low ion beta 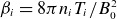 , where such effects (for the ions) are typically relatively weak since the typical phase velocity 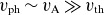 . Note we have also assumed that the electric and magnetic fields do not vary in the 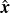 direction. We assume that the functions 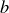 and 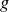 are analytic for 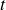 real and that 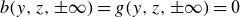 . Here, 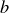 and 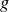 are related according to Faraday’s law,
(2.3)

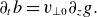

We carry out our calculation in the frame moving at 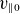 , the parallel velocity of the ion at 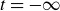 , and normalise according to
(2.4)

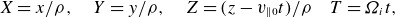

where 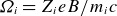 is the ion gyrofrequency and 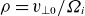 is the ion gyroradius. Later, it will be useful to write 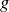 and 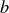 in terms of their Fourier transforms in 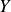 ,
(2.5)

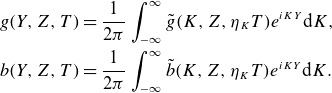

The dimensionless quantity 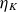 appearing in the arguments of 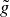 and 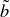 is a bookkeeping parameter that describes how fast the fields at wavenumber 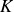 vary relative to the cyclotron motion of the particle: for 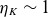 , the fields can vary significantly over one orbit, while for 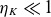 , they only vary a small amount. Importantly, we do not require 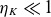 : in fact, for the main calculation that appears in § 3, we formally require 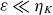 for all 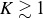 , i.e. 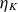 cannot be too small. The case with 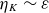 or smaller is dealt with in Appendix C, where we show that our results can be extended to this case with no changes. If 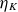 in some system happens to be constant with 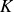 , we will sometimes simply write 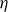 . Denoting 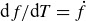 , the equations are then
(2.6)

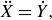



(2.7)

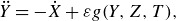



(2.8)

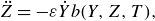

which we will solve subject to the arbitrary choices for the phase of the particle 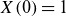 , 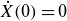 , 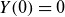 , 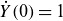 , 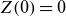 , and we have chosen the inertial frame of reference such that 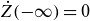 . In the normalised variables, Faraday’s law is
(2.9)

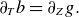

Integrating (2.6), taking the constant of integration to be zero and inserting the resulting equation into (2.7), we have
(2.10)

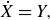



(2.11)

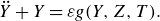




## Solution for 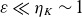


3.

We expand
(3.1)

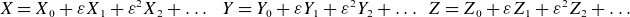

and proceed with our calculation.^1^ At zeroth order in 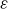 , we just have the gyration of the particle about the background field,
(3.2)

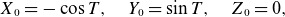

according to the (arbitrary) conditions we set for 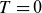 . At first order in 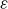 , inserting the zeroth-order solution above for 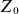 and 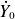 into (2.11) and (2.8),
(3.3)

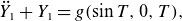



(3.4)

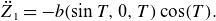

Equation (3.3) may be solved by Fourier transforming in time and back again; the solution is
(3.5)

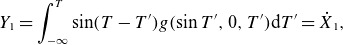

and the first-order 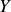 -velocity is
(3.6)

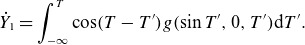




To make further progress, we Fourier transform in 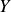 according to (2.5), and use the identity
(3.7)

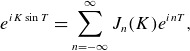

where 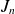 are Bessel functions of the first kind. This results in
(3.8)

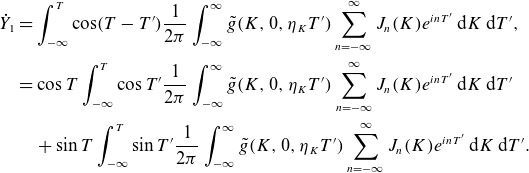




Similarly, we have
(3.9)

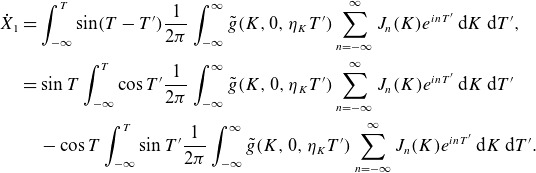




Combining the sinusoids and 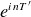 factors in the integrands, and using the identity 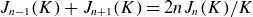 ,
(3.10)

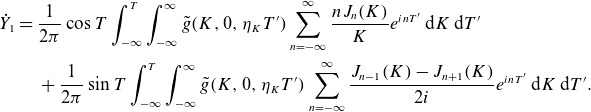




Likewise, one finds
(3.11)

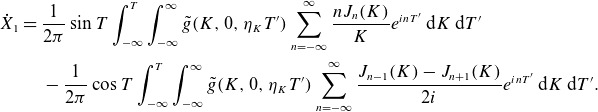

Finally, using (2.5) to Fourier transform 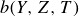 , the first-order parallel velocity is given by
(3.12)

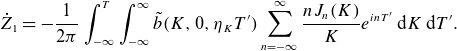




### 3.1. Change in perpendicular energy

We are interested in the change in 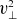 as 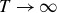 ,
(3.13)

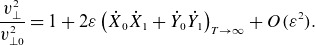

Using (3.2) differentiated with respect to 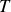 , (3.11) and (3.10),
(3.14)

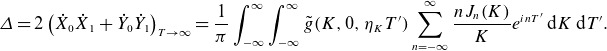




Clearly, the contribution from 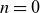 in the sum vanishes. The integral is of the form
(3.15)

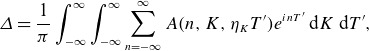

which we can perform by closing the contour in the appropriate half-plane. The dominant contribution comes from the pole of 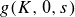 (say 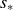 ) closest to the real axis, so that
(3.16)

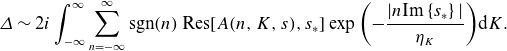




Since 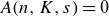 for 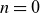 , if we have that 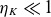 for all 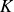 , this is exponentially small. At higher order in 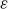 , similar exponentially small expressions occur; this is a special case of the general conservation of adiabatic invariants to all orders (Kruskal 1958, 1962).

Moreover, if 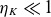 for all 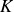 , we need only keep the 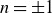 term, since it is obviously the largest. Therefore, we approximate 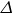 as
(3.17)

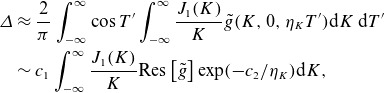

where 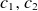 are (system-dependent) dimensionless constants of order unity and 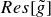 denotes the residue from the pole of 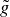 closest to the real axis, and is a function of 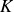 .

At this point, it is worth discussing when and how our solution breaks down. First, note that it is possible to have a situation where the exponential term arising from the pole is cancelled out by part of 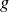 : for example, if 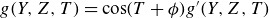 . This is resonance and leads to the breakdown of the ordering if 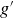 is non-zero for a time 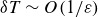 . We will assume this is not the case, but briefly discuss it in Appendix B.

Second, while we have shown that the first-order change in perpendicular kinetic energy (3.14) is exponentially small for 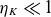 for all 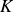 , the same is not true for our expressions for the perpendicular velocities (see (3.10)–(3.11)): the second integral in each case clearly has a non-zero 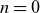 term, meaning that if the fields are left on for a time 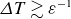 , the ordering of the solution will break down due to secularly growing terms in 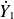 and 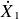 . This could be the case, for example, if the field varies so slowly that 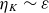 ; hence, our formal restriction to larger 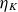 in this section. These secular terms cancel out in the expression (3.14) for the change in kinetic energy. Unlike the case of a true resonance, this is simply a consequence of the naive perturbation method. In Appendix C, we use the Poincaré–Lindstedt method to extend the calculation to the case of arbitrarily small 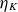 , showing that the expression (3.14) for the change in perpendicular kinetic energy does not change. This more involved calculation is therefore perhaps of more mathematical than physical interest, but is included for completeness.

### 3.2. Scale dependence

Because of the Bessel function, the contributions to 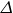 from different perpendicular scale 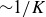 vary with 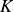 . For small argument ( 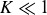 ), 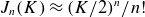 , so that the only term that survives in (3.10) is 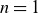 , and we may replace 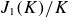 in (3.17) with a scale-independent factor 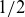 . In the opposite limit of large argument 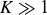 , the envelope of 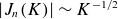 , and so all the terms in (3.8) become small. However, in turbulence, 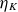 is typically an increasing function of 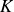 , and the exponential suppression of the heating will be less effective for larger 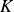 : the balance between these is system-specific, depending on 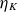 and the 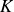 -dependence of the Fourier amplitudes 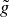 .

### 3.3. Scattering contours

Let us for the moment assume that the electromagnetic fluctuations are from a propagating wave or superposition of waves, with a parallel phase velocity 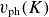 , so that 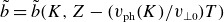 and 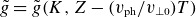 . The results derived in the previous sections do not require this, but it will allow us to make contact with the usual quasilinear theory of cyclotron heating. It may also be directly applicable to the turbulence in the solar wind, which can be highly imbalanced: dominated by outward-going Alfvén waves, for which 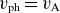 . More generally, this situation could potentially also apply to nonlinear solitary waves which have a single effective 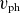 due to the nonlinearity balancing dispersion (Kawahara 1969; Kakutani & Ono 1969; Hasegawa & Mima 1976; Mjølhus & Wyller 1986; Mallet 2023). We note that we are ignoring the possibility of waves purely with phase velocity only in the perpendicular direction, and also electrostatic waves (which to have parallel phase velocities, must also have 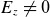 so that Faraday’s law is satified).

In a frame moving at 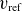 compared to the laboratory, to first order in 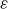 , as 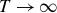 , we have the energy change
(3.18)

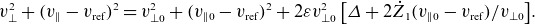




From Faraday’s law (2.9), we have
(3.19)

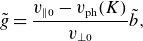

so that (3.14) can be written as
(3.20)

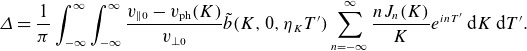




Combining this expression with (3.12) as 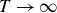 , we find that
(3.21)

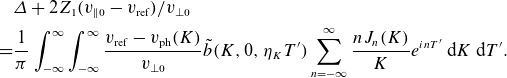




The integrand of this expression vanishes if 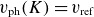 . The left-hand side is the expression appearing in square brackets in (3.18). Therefore, for a propagating wave or coherent wavepacket (e.g. a soliton), diffusion occurs along the scattering contours 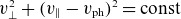 . This behaviour is lost if there is not a single 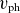 , as would be the case for a dispersive wavepacket where 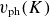 is not constant. This is also the case in strong, balanced Alfvénic turbulence, where while the linear and nonlinear frequencies are statistically in critical balance, so that 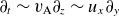 , there is a broad range of effective frequencies; or equivalently, a distribution of effective phase velocities with mean zero and width 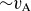 .

Obviously, for a particle moving at 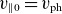 , the electric field is zero (again, provided that there is no electrostatic wave), the magnetic field is stationary in time and, thus, there is no change in the perpendicular or parallel energy of the particle. This is encoded in the fact that for a particle moving at 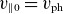 , the phase of the wave is 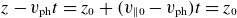 , independent of 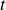 , and so 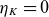 for all 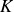 .

### 3.4. Example

Let us (for simplicity’s sake) assume that 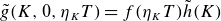 . As an example, we choose
(3.22)

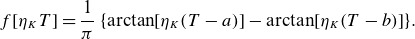

**Figure 1.**
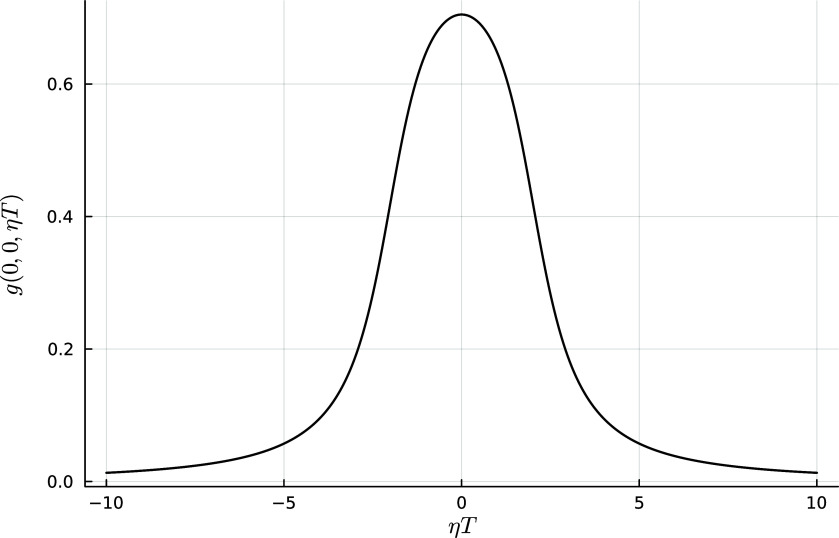
The functional form (3.22) for the time dependence of the electric field, with 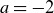 , 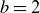 .

We will leave the spatial pattern of the fluctuation 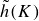 arbitrary since we are mainly interested in the dependence of the energy change on 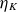 . This is a model of a fluctuation that ‘turns on’ at a rate 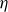 around 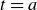 and then ‘turns off’ at the same rate at 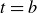 , and is plotted in figure 1. We need to perform integrals of the form
(3.23)

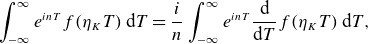

for integer 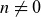 , and we have integrated by parts to get the second expression.^2^ We have
(3.24)

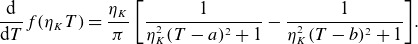




The poles are at 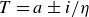 , 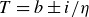 . We close in the appropriate half-plane, and obtain for 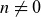 ,
(3.25)

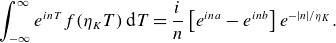




Then, the perpendicular energy change (3.14) is
(3.26)

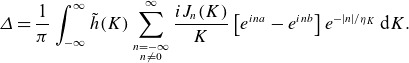




Two simplified limits are of interest: first, if 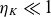 and the fields vary slowly compared with the gyrofrequency, the 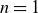 terms dominate (and even they are exponentially small):
(3.27)

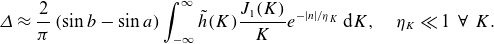




Second, if 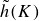 only has power at small 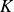 , we can again drop all but the 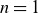 term, as discussed earlier in § 3.2, and additionally 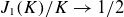 :
(3.28)

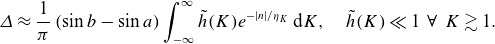




We can understand the dependence on 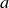 and 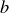 as depending on the phase of the particle’s orbit at some reference time. Assuming that the ion velocity distribution is gyrotropic, each individual particle is as likely to gain or lose energy from the interaction: the average 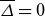 .

### 3.5. Diffusion coefficient and heating rate

We have so far derived an expression for the change in perpendicular energy of a single particle, 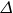 (see 3.14) and derived its explicit form for an example (3.26). Importantly, 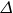 can be both positive and negative, and in fact, the average over the initial gyrophase of the particle 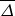 vanishes.

Repeated interactions with coherent fluctuations will cause diffusion in energy. To be more precise, let us suppose for the moment that there are a large number of identical coherent fluctuations present and the particle encounters one approximately every 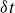 . Each interaction with a fluctuation occurs with a random initial gyrophase and provides an uncorrelated kick in perpendicular kinetic energy of magnitude 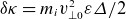 . This leads to an energy diffusion coefficient
(3.29)

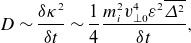

where the overline denotes averaging over the (uniform) gyrophase distribution of the particles.

If all the fluctuations are characterised by a single perpendicular scale 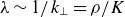 ,^3^ then the normalised time scale for the interaction is 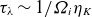 . Let us now suppose that these fluctuations are rare: in each time interval of length 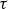 , an encounter with a fluctuation occurs with probability 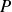 . Then, the 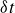 appearing in the formula for the diffusion coefficient is clearly just 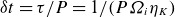 , i.e.
(3.30)

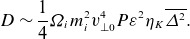




Now, consider the more realistic case where at each scale 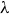 , there is an ensemble of different fluctuations, each with their own 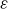 , 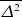 and 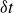 : we may characterise this ensemble by a joint probability distribution 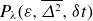 , noting that the arguments need not be independent. Then, we can generalise (3.30): denoting the average over the distribution of fluctuations 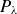 with angle brackets,
(3.31)

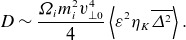

In the case of fluctuations that are propagating (linear or nonlinear) waves, so that 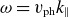 , the diffusion will be along the scattering contours (§ 3.3).

Finally, we can use our expression (3.17) for 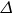 to estimate
(3.32)

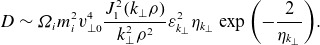

As 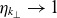 from below, the diffusion becomes strong. To get an overall effective heating rate per unit mass, suppose 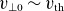 ; then,
(3.33)

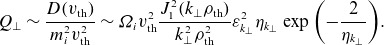




We have derived this diffusion coefficient for the energy of a single particle interacting with a distribution of fluctuations. If we consider the whole population of ions, with ion velocity distribution function 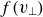 , interacting with a single coherent fluctuation, the behaviour is also diffusive. If there is a gradient in 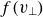 , while individual particles are just as likely to gain or lose energy, the flux of particles from the region with larger 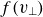 will be larger than the flux from the region with smaller 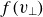 , smoothing the gradient. As an illustration, suppose that the initial distribution is uniform in gyrophase, but confined to a single 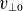 : a ring distribution. Afterwards,
(3.34)

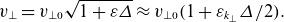

The variance of this distribution is then
(3.35)

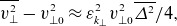

i.e. the effective temperature has changed by an amount of order
(3.36)

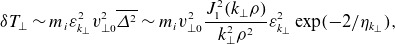

where we have used (3.17) to estimate 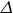 . As 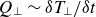 and 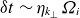 , (3.33) and (3.36) agree with each other.

In the rest of the paper, we will use the theory already described to study ion heating in Alfvénic turbulence (using (3.33), since over a long time period, each particle will interact with many fluctuations) and reconnection (using (3.36), since the particles only interact with a reconnection exhaust once). To do so, it is necessary to have on hand estimates of 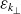 and 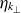 , the accuracy of the latter being more critically important: due to its presence inside the exponential cutoff, our cavalier disregard of coefficients of order unity might lead to large inaccuracies in the estimated heating rates. For this reason, in much of the rest of the paper, we will (following the approach of Chandran *et al*. 2010) insert adjustable constants parametrising these unknown coefficients in our estimates for 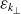 and 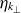 . Given a detailed enough knowledge of the system’s dynamics, it would in principle be possible to derive these coefficients from first principles; more practically, one can fit them numerically (Xia *et al*. 2013; Cerri *et al*. 2021; Johnston *et al*. 2025), although care must be taken to consider the unrealistically limited scale separation possible in simulations of turbulence and reconnection.

There are a few different approaches to estimating 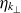 . In the first, we estimate 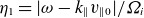 , where 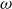 is some linear or nonlinear frequency of the system. If 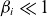 and we have Alfvénic fluctuations with 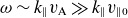 , the 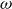 term tends to dominate. In the second, we estimate 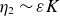 as the (inverse of the) time it takes to 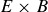 drift out of the structure, assuming that, in reality, the fields have structure in the 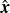 direction too. This can only possibly be relevant once 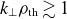 , since for 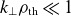 , the fields are frozen into the plasma flow.

Finally, one might think to estimate the time it takes for the polarisation drift ( 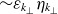 ) to cause the particle to leave the structure in the 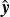 direction (McChesney *et al*. 1987; Chen, Lin & White 2001; White, Chen & Lin 2002), 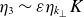 , where probably 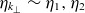 . As can be seen, this is only comparable to 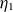 or 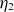 if 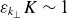 , i.e. only at very large amplitude.

To preview the approach of the next two sections, in Alfvénic turbulence (§ 4), we will find that 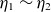 , while in reconnection (§ 5), we will use 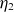 exclusively, assuming little structure in the parallel direction.

## Low- 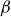 Alfvénic turbulence

4.

In Alfvénic turbulence is present in the solar wind and corona, both 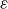 and 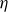 depend on 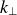 . We will assume a relatively low 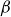 , so that the kinetic reduced electron heating model (KREHM) equations (Zocco & Schekochihin 2011) may be used; we also assume that while 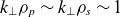 , 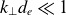 , so the electrons are isothermal; where the thermal proton gyroradius 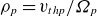 (different from 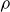 !), the ion sound radius is 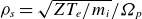 , with 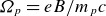 the proton gyrofrequency, and 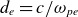 is the electron inertial length. We want to express our results in terms of what is experimentally observable in the solar wind; namely, the 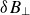 fluctuation amplitude as a function of the perpendicular wavenumber 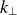 ; we will write this in velocity units as 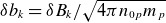 . For now, we will also neglect intermittency in the turbulent fluctuation amplitude, supposing that we may characterise the amplitude at each scale by a single value 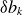 . (The effects of intermittency will be examined in § 4.4.) Moreover, we assume the critical balance, such that 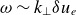 , where the effective electron bulk flow velocity is (Zocco & Schekochihin 2011)
(4.1)

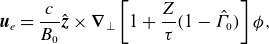

where 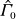 is the inverse Fourier transform of
(4.2)

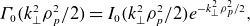




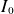
 being the modified Bessel function. The 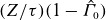 appearing in the square brackets on the right-hand side of (4.1) results from the diamagnetic drift; the electron density fluctuations are 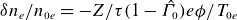 . For Alfvénic fluctuations, one finds that 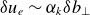 , where
(4.3)

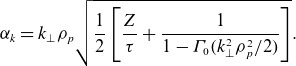

This is true not only for linear Alfvén waves, but statistically even in strongly nonlinear kinetic-Alfvén turbulence (Grošelj *et al*. 2018): in a similar sense to the fact that 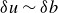 in strong MHD turbulence (Maron & Goldreich 2001). Since 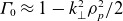 for 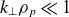 , but 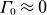 for 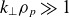 , 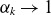 as 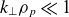 (Alfvén waves) and 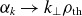 for 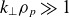 (kinetic Alfvén waves). Thus, we have
(4.4)

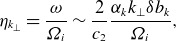

where we have neglected 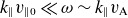 since 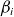 is small and, as promised, added an undetermined constant 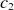 accounting for (several) prefactors of order unity we have neglected. This could, in principle, be corrected for the slowing down of the turbulent cascade due to dynamic alignment (Boldyrev 2006; Chandran *et al*. 2015; Mallet & Schekochihin 2017) and/or imbalance (Schekochihin 2022; Chandran *et al*. 2025). A more important limitation is that the KREHM equations assume that 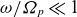 . We will rather flagrantly ignore this restriction in the following analysis: while it could be corrected for by using a more accurate dispersion relation, we believe it does not impact our results in a significant way. The parameter 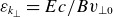 controlling the amplitude of the electric fields is similarly found:^4^ using 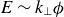 , and using (4.1) and (4.3),
(4.5)

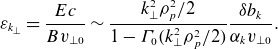

**Figure 2.**
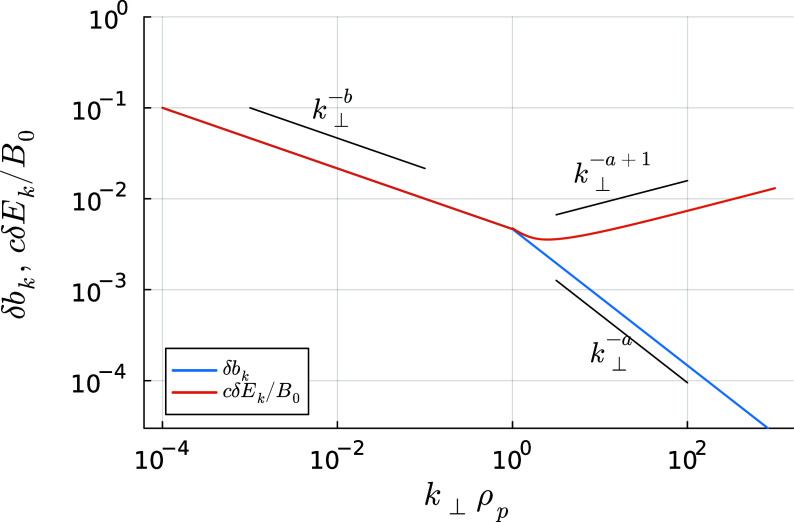
Typical scalings for the magnetic and electric field fluctuation amplitudes as a function of 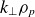 , in the absence of strong dissipation. Here, we have set 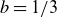 and 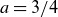 , and the sub-ion-scale range is unrealistically long: in reality, it would be cut off at the smaller of 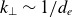 or 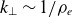 . We do not model electron-scale effects in this paper.

The dependence of the electric field fluctuations on 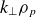 is quite different from the magnetic field fluctuations. At 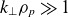 , 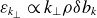 , as can be seen in the example spectrum plotted in figure 2. This means that, in the absence of dissipation, the electric field fluctuations increase with 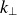 for 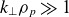 , as observed in fully kinetic turbulence simulations (Grošelj *et al*. 2018). Putting this all together with the estimate for the perpendicular diffusion coefficient (3.32),
(4.6)

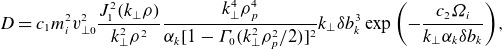

where the order-unity constants 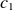 and 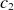 account for all the numerical prefactors as well as ‘twiddles’ ( 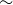 ) appearing in our previous estimates. Using (3.33), the heating rate for thermal ions (i.e. assuming 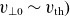 is then
(4.7)

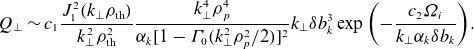




For the moment, assuming that the ion component of the plasma is mainly protons,^5^ 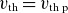 , 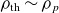 and 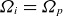 , we have
(4.8)

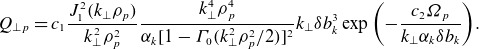

We will return to the subject of minor ions in § 4.5. Taking 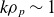 , we recover the expression given by Chandran *et al*. (2010), where the exponential suppression factor depends only on the amplitude of the fluctuations: the equivalence between exponential suppression factors based on the time scale and the amplitude for 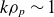 was first noticed by Cerri *et al*. (2021). However, we can now assess the scale dependence of the heating directly. It is interesting to look at this in different limits. For 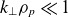 , we have 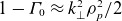 , 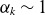 and 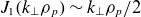 . Then,
(4.9)

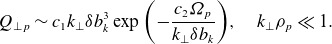

For 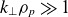 , 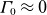 , 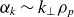 (ignoring dependence on 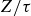 for simplicity) and the envelope of 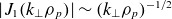 , so that
(4.10)

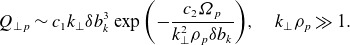




The only difference is in the exponential suppression factor; the speedup in the frequency for small-scale fluctuations means we get an extra factor of 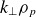 there. Were 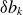 independent of scale, the heating rate becomes monotonically larger towards smaller scale, since the frequency increases. Equations (4.8)–(4.10) can be compared with the heating rate (due to Landau/transit-time damping) in Alfvénic turbulence within the gyrokinetic model (Quataert 1998; Howes 2010), 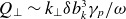 : since 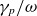 is rather small at low 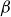 , magnetic moment breaking may be the dominant heating mechanism despite the exponential suppression factor. Future work will assess this statement in a more satisfactory manner.

### 4.1. Balanced turbulence

We will first examine the case of ‘balanced’ turbulence, where the fluxes of Alfvénic fluctuations propagating parallel and antiparallel to the magnetic field are comparable: the imbalanced case is rather different and is discussed in § 4.3. Typical scalings for the fluctuation amplitudes 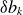 and 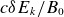 in balanced turbulence are plotted in figure 2. At small scales, 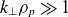 , we have 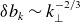 or steeper (Schekochihin *et al*. 2009; Boldyrev & Perez 2012; Zhou, Liu & Loureiro 2023); say 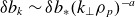 , where 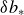 is then the amplitude at 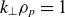 . Then,
(4.11)

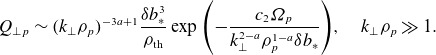




This reaches a maximum when
(4.12)

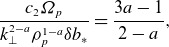

or at
(4.13)

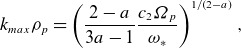

where 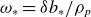 is the frequency of gyroscale fluctuations. The maximum proton heating rate therefore occurs when 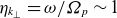 (in an order-of-magnitude sense). Here, we remind the reader that in reality, this estimate will become inaccurate since our estimates will fail around 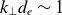 , where the spectrum steepens again (Stawarz *et al*. 2019) and our estimates for 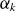 also break down (Adkins, Meyrand & Squire 2024).

**Figure 3.**
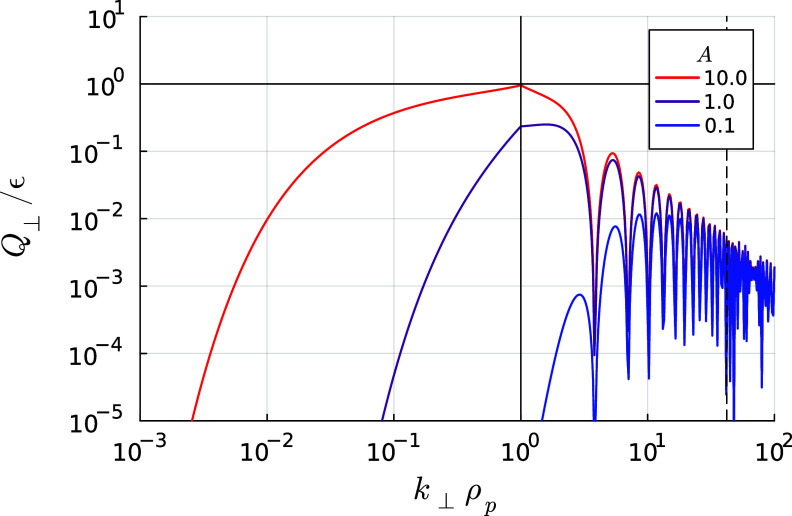
The proton heating rate 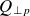 normalised to the turbulent energy flux through scales 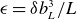 for different values of the normalised outer-scale amplitude 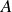 , with 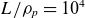 and 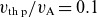 . The horizontal black line denotes 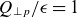 , complete damping of the turbulent cascade: in reality, if the heating approaches this line, the power-law behaviour of the spectra and the constancy of 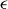 will no longer be accurate. The vertical solid black line denotes 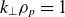 and the vertical dashed line denotes 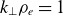 : the small heating rates at or beyond the electron scales in our model (which neglects electron-scale physics) are an overestimate due to the much steeper spectrum in this range.

At large scales, 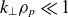 , the magnetic fluctuations scale as a power law, between 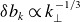 (Goldreich & Sridhar 1995; Chen *et al*. 2011) and 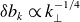 (Boldyrev 2006; Mallet *et al*. 2016; Chen *et al*. 2020), where the exact scaling likely depends on the regime of turbulence. Then, 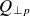 is an increasing function of 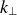 . In balanced turbulence, there is typically a smooth join between the 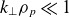 and 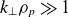 spectra, and so we expect the heating rate as a function of 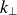 to reach a maximum at relatively small scales, when 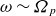 . This agrees (qualitatively at least) with the peak of the heating rate observed in the hybrid-kinetic numerical simulations of Arzamasskiy *et al*. (2019) and Cerri *et al*. (2021). We have plotted the proton heating rate (4.8) as a function of (inverse) scale 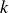 in figure 3 for several different outer scale amplitudes 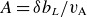 , assuming that 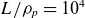 and 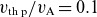 . The energy flux into the turbulent cascade is defined as 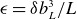 . The trend agrees with our previous analysis: if the amplitude is high enough that 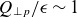 at 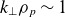 , the peak heating is at the proton gyroradius scale (e.g. the red line in figure 3). At lower amplitudes, the heating is less efficient and peaks at a smaller scale (e.g. the blue line in figure 3). The oscillations in 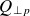 when 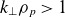 are due to the Bessel function; in reality, structures will have contributions from a range of 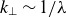 and this behaviour will be smoothed out.

### 4.2. Proton heating fraction in balanced turbulence

The maximum proton heating rate is
(4.14)

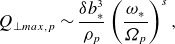

ignoring prefactors of order unity, and with 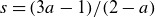 . Now, suppose the turbulent cascade (in the absence of dissipation) had a constant energy flux through scale 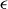 ; by a Kolmogorov-style argument, dimensionally (as a reminder, we are neglecting intermittency in the distribution of fluctuation amplitudes),
(4.15)

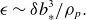

If 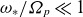 , 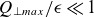 and there is no significant ion heating; the energy must be dissipated at smaller scales onto the electrons. Writing 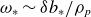 ,
(4.16)

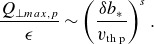




With 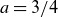 (a not unreasonable value given the observed spectrum, e.g. Chen *et al*. 2010), 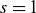 and this agrees with the expression for the ion heating fraction given by Matthaeus *et al*. (2016). It may be more useful to write this in terms of the amplitude at the outer scale 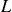 of the turbulence, parametrised as
(4.17)

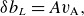

where 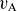 is the Alfvén velocity. Assuming 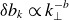 for 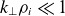 (with 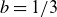 corresponding to Goldreich & Sridhar 1995 and 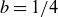 corresponding to Boldyrev 2006 including dynamic alignment), we have
(4.18)

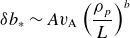

and
(4.19)

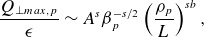

where the proton plasma beta 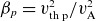 . Inserting 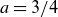 ( 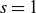 ) for simplicity,
(4.20)

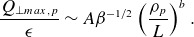

This estimate can be compared with other mechanisms, for example, with the approach outlined by Howes (2024): for a dissipation mechanism to be important, we require the turbulent system to pass a threshold in parameter space beyond which 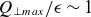 . Here, this threshold is described in terms of 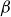 and 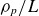 . For perpendicular ion heating to be important, we need (taking 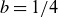 )
(4.21)

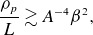

which is the same dependence on 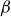 as found by Howes (2024) for stochastic heating (Chandran *et al*. 2010). Typically, 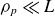 : for example, in the solar wind, 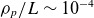 , while in the interstellar medium (ISM), 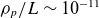 . Thus, this simple estimate suggests that ion heating should be negligible in the ISM, and accounts for only 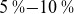 of the energy budget in the 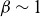 solar wind, in stark contrast with the available evidence (Cranmer *et al*. 2009). This suggests that we need to incorporate additional physics into our model: in § 4.4, we show that intermittency (Mallet *et al*. 2019) results in much higher ion heating fractions, because fluctuations attain larger amplitudes such that 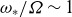 , leading to 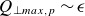 .

We have neglected all heating apart from that at 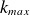 : while amending this might increase the heating rates slightly, in practice, because of the exponential suppression of heating from low-frequency fluctuations, 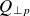 is quite sharply peaked at 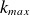 .

### 4.3. Forced imbalanced Alfvénic turbulence and the helicity barrier

Meyrand *et al*. (2021) found that in numerical simulations of forced imbalanced turbulence, the helicity barrier causes energy to build up at scales larger than 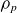 with a spectral break to a very steep ( 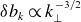 or steeper) spectrum beyond 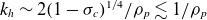 in the ‘transition range’, before returning to a shallower spectrum at 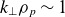 . We can study this situation with our heating rate expression (4.9) also. Writing 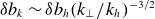 and taking 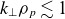 , the frequency 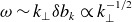 in the transition range, as a decreasing function of 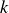 . Inserting into our expression for 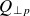 for 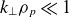 , we find
(4.22)

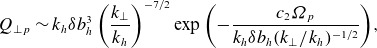

which decreases with increasing 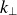 in the transition range. The assumption 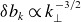 could be weakened; 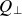 always decreases with 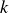 so long as 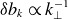 or steeper. Thus, the maximum heating occurs at 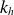 and is given by
(4.23)

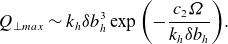

If 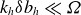 , then 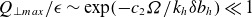 . If energy is being injected into the turbulent cascade at large scales, this situation cannot be in steady state due to the presence of the helicity barrier, and thus 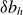 (and the whole large-scale spectrum) must be growing in time. A steady state can be achieved once 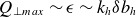 , which happens when 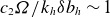 , or roughly 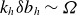 . Thus, in forced imbalanced turbulence, essentially all the energy flux from the turbulent cascade goes into ion heating. A more refined analysis, taking into account the electron inertia effects entering at 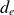 , shows that in fact there is a critical level of imbalance below which the helicity barrier will not form (Adkins *et al*. 2024): we assume that in the systems in which we are interested (for example, the solar corona), 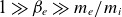 and the turbulence is extremely imbalanced, such that we can ignore this effect.

This picture of ion heating ‘switching on’ due to the helicity barrier is, as we mentioned previously, not new, and already predicted by Meyrand *et al*. (2021) and Squire *et al*. (2021). Even more similar to this work, Johnston *et al*. (2025) found using test particle simulations that ion heating in imbalanced turbulence depends on a phenomenological exponential suppression factor, controlled by the fluctuation amplitude at the scale at which the maximum frequency is reached, i.e. the above-mentioned transition-range break scale 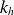 .

Physical systems of imbalanced turbulence, for example, the solar wind, are not in fact homogeneous nor forced in the usual way, and so the helicity barrier may not have sufficient time to reach steady state and cause as sharp a transition to ion heating as suggested based on the forced numerical simulations (Meyrand *et al*. 2021; Squire *et al*. 2021). The scaling for the maximum heating rate (4.23) still applies, since the helicity barrier does still cause a steep transition range at scales larger than 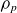 .

### 4.4. Intermittency

So far, we have assumed that the turbulence is characterised by a single amplitude at each scale, 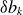 . In reality, 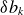 is a random variable at each scale, typically with a heavy large-amplitude tail; both in solar wind observations (Salem *et al*. 2009; Zhdankin, Boldyrev & Mason 2012; Sioulas *et al*. 2022) and in numerical simulations (Mallet *et al*. 2015, 2016; Zhdankin *et al*. 2016*a*, *b*). This can dramatically increase the ion heating fraction (Mallet *et al*. 2019; Cerri *et al*. 2021).

As an (extreme) toy example, suppose that the turbulence were characterised by fluctuations of a fixed amplitude 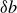 , filling a certain scale-dependent fraction 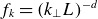 of the volume at scale 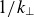 . In other words, the distribution of fluctuation amplitudes is
(4.24)

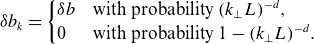

Requiring the energy flux 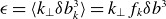 to be independent of 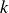 , we have 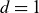 . Then, the root-mean-square value of the fluctuation amplitude measured at scale 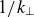 is
(4.25)

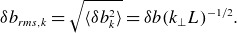

Meanwhile, the overall heating rate at large scales, given by (4.9), is
(4.26)

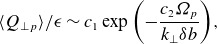

where the average is over the distribution of fluctuation amplitudes. Writing this solely in terms of 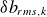 ,
(4.27)

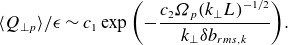




Since 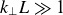 , this dramatically increases the overall heating rate for a given observed 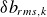 , compared with the estimate without taking account of the intermittency of 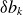 . This is not, in fact, a realistic model of intermittent Alfvénic turbulence – we have included it here to show, in a transparent way, the difference that intermittency makes to the efficiency of ion heating.

It is worth mentioning that intermittency models including dynamic alignment (e.g. Chandran *et al*. 2015; Mallet & Schekochihin 2017) do not necessarily increase the ion heating fraction, because the dynamic alignment between 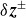 increases the nonlinear time scale of the turbulent structures, decreasing the effectiveness of the heating due to the exponential suppression factor. This decrease in ion heating fraction due to dynamic alignment is the opposite to what was found earlier by Mallet *et al*. (2019), who used an exponential suppression factor based solely on the amplitude (Chandran *et al*. 2010), rather than the rate of change of the fluctuations, thus finding that dynamic alignment acted to increase the stochastic heating rate.

A more promising intermittency model in this regard (which nevertheless obtains the same 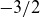 perpendicular spectrum) is the reflection-driven turbulence model of Chandran *et al*. (2025), in which dynamic alignment does not play a role. There, larger-amplitude fluctuations naturally have higher frequencies, as required to make the ion heating more effective. Another approach, independent of any particular intermittency model, would be to use *in situ* measurements of the distribution of turbulent fluctuations to calculate the heating rate 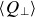 directly. The Parker Solar Probe has recently started to explore the plasma environment very close to the sun (Kasper *et al*. 2021), where ion heating is thought to be particularly important (Kasper & Klein 2019), and it will be interesting to assess the ion heating in this newly explored regime.

### 4.5. Minor ions

Observationally, as mentioned in § 1, minor ions appear to be heated even more strongly than the protons. Returning to (4.7) and writing the ion gyrofrequency in terms of the proton gyrofrequency, 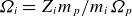 ,
(4.28)

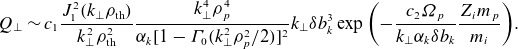




Since 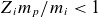 , the exponential suppression is less effective for minor ions, exponentially increasing the heating rate relative to the protons. This is similar to what happens in the stochastic heating model applied to minor ions (Chandran 2010). Note that 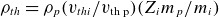 , so that the gyroradius of the ions is typically larger than that of the protons: however, in the case of imbalanced turbulence where the cascade is already cut off due to the helicity barrier (§ 4.3), this may have less of an effect. An exception is in forced homogeneous numerical simulations, where the saturated state of the turbulence has such a high amplitude that fluctuations attain the minor ion’s gyrofrequency at large scales, causing extreme heating of minor species (Zhang *et al*. 2025).

## 5. Reconnection

**Figure 4.**
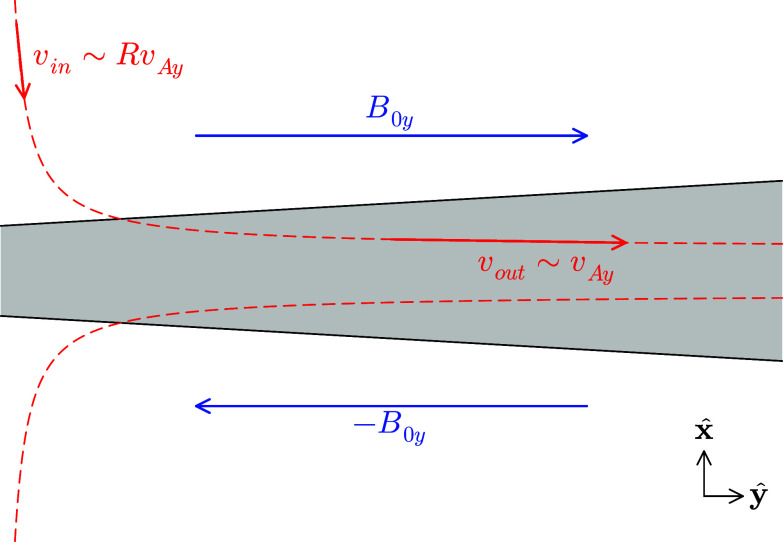
A crude schematic of an ion-scale reconnection exhaust. The reconnecting field 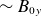 (blue) reverses across the exhaust. An ion enters the exhaust with a slow drift velocity (red) 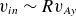 with the reconnection rate 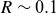 and 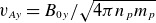 . Within the exhaust, due to the strong electric field 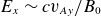 , where 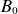 is the guide field, the ion takes up a drift at the Alfvénic outflow velocity 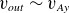 . If this process happens in a time comparable to the ion’s gyroperiod, the magnetic moment is not conserved and strong heating occurs.

Drake *et al*. (2009*a*) developed a theory of perpendicular ion heating in reconnection and Drake *et al*. (2009*b*) showed that for guide-field reconnection, there was a threshold for strong ion heating by different ion species, namely
(5.1)

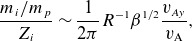

where 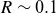 is the normalised reconnection rate 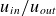 , 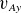 is the Alfvén speed based on the reconnecting field 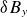 , and 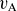 is the Alfvén speed based on the guide-field 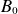 . This was derived in the following way (see figure 4 for a schematic illustration of this scenario). Given a current sheet width of order 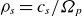 and an inflow speed 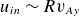 , the transit time of an ion from the inflow out of the sheet is of order 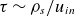 . Comparing this with the ion’s gyrofrequency 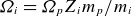 and requiring 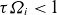 gives (5.1). Below the threshold, the ions conserve the first adiabatic invariant 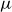 , while above the threshold, 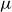 is not conserved and strong ion heating occurs. There is an obvious equivalence between the threshold in 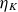 in our estimated heating rate (3.33) and the theory of Drake *et al*. (2009*b*). To make this more concrete, we note that in the current sheet, there is an 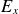 driving the Alfvénic exhaust,
(5.2)

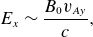

from which we estimate 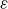 ,
(5.3)

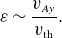

For 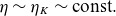 (we assume this does not vary with 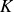 since the outflow is coherent in time), we use the same argument as Drake *et al*. (2009*a*), leading to
(5.4)

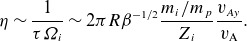




Applying (3.36) and estimating 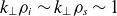 so that the Bessel function term is of order unity,
(5.5)

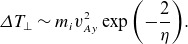

Apart from numerical prefactors of order unity which we have not calculated, this agrees with (6) of Drake *et al*. (2009*a*), both in the threshold 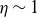 and in the order-of-magnitude of the saturated total heating when the threshold is attained. It would also technically be possible to calculate the heating at a reconnection event using our model by specifying the functional form of the electromagnetic fields precisely, arriving at a more accurate estimate; we will leave this for future work.

This section amounts in some ways to a rederivation of the model of Drake *et al*. (2009*b*). However, using our approach, it is perhaps more obvious why the acceleration of the ion into the outflow 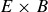 velocity is accompanied by ion heating. Drake *et al*. (2009*a*) suggest that ‘the reflected particles interpenetrate with particles that have already crossed the boundary of the exhaust but have not passed through the reversal region’, thus gaining an effective temperature. In our approach, it is obvious that the diffusion is due to the random initial phase of the particle as it enters the reversal region of the exhaust. While the energy change of each ion 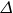 is, on average, zero, considering the plasma distribution function as a whole, the ions acquire a broad range of energies.

## 6. Discussion

We have analysed the interaction of an ion with a localised, coherent fluctuation, deriving the change in the ion’s perpendicular and parallel kinetic energy. To lowest order, the energy change is described by (1.1), which depends linearly on the amplitude of the fluctuating fields and on a factor 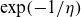 , where 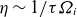 , with 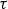 the characteristic time scale of the fluctuation and 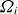 the ion’s gyrofrequency. For the whole population of ions, the interaction leads to diffusion in energy and heating. This leads to weak heating for 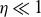 and strong heating for 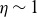 : this reflects the well-known conservation of the magnetic moment for 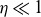 and is similar to previous theories of stochastic heating (Chandran *et al*. 2010). As part of our derivation, we have recovered the fact that, if the fluctuation is a wavepacket with a fixed phase velocity 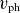 , the diffusion is along circular scattering contours in 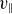 – 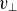 space, centred on 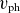 (§ 3.3), a result usually associated with quasilinear cyclotron resonant heating (Kennel & Engelmann 1966). Thus, our results combine the physics of stochastic heating and cyclotron heating in a single theoretical framework, based on the interaction of ions with coherent structures. In many systems, our approach based on individual localised fluctuations may be more appropriate than the assumption of infinite plane waves usually required to derive the results of quasilinear theory, for example, in strongly nonlinear turbulence and reconnection. The model is also quite easy to apply to different physical situations: one needs estimates for a characteristic time scale relative to the gyroperiod, 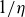 , as well as an estimate of the fluctuation amplitude 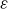 of the structure.

We have applied our results to low- 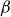 Alfvénic turbulence, obtaining a simple expression for the fraction of the turbulent flux absorbed by the protons in both balanced turbulence and in imbalanced turbulence with a helicity barrier (Meyrand *et al*. 2021), as may be present in the fast Alfvénic solar wind (Bowen *et al*. 2020; McIntyre *et al*. 2024). Our theoretical estimate for the proton heating fraction compares well with the numerically observed estimate for the ion heating fraction of Matthaeus *et al*. (2016). We also show that our model is well suited to incorporating different models of intermittency, which we predict should enhance the ion heating: one could also use the observational data to assess this enhancement directly. Moreover, we have determined how the perpendicular length scale of the fluctuation affects the heating rates: at smaller amplitudes, the heating does not peak at 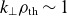 , but at a smaller scale, as was previously observed in the hybrid-kinetic simulations of Arzamasskiy *et al*. (2019). We also show that the heating of minor ions is greatly enhanced over the proton heating, another property associated with both cyclotron (Kasper *et al*. 2013) and stochastic (Chandran 2010) heating. Our framework can also model ion heating in reconnection, naturally producing perpendicular heating with a threshold similar to the theory and numerical simulations of Drake *et al*. (2009*a*, *b*). The similarity of our results for heating due to reconnection and turbulence suggest that the disparity between these two paradigms for coronal heating (Chandran & Hollweg 2009; Klimchuk 2015) may not be as drastic as often thought.

Johnston *et al*. (2025) have shown within the framework of quasilinear theory that it is possible to recover an exponential or exponential-like suppression of heating, similar to our model and to the original stochastic heating theory of Chandran *et al*. (2010). Moreover, they show that test-particle heating in both balanced and imbalanced turbulence simulations seems to be well described by such an exponential suppression factor. As reported both by Johnston *et al*. (2025) and in this paper, the exponential factor depends on the typical frequency or inverse time scale of the interactions compared with the ion cyclotron frequency, i.e. 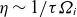 , reinforcing the fact that the relevant physics in both cases is the breaking of the magnetic moment conservation. However, the source of the suppression factor is different: Johnston *et al*. (2025) reported that the suppression factor in imbalanced turbulence is due to the steep 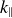 spectrum outside the critical balance cone leading to very little power at the high resonant frequency, whereas in our case, it comes from the fact that 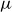 is an adiabatic invariant as 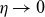 , conserved to all orders.

## Footnotes

1 As mentioned previously, in this section, we formally require that the fields do not vary too slowly at small scales:  for all  ; this does not preclude  , so long as  . We will point out clearly where the calculation breaks down for the case of  and in Appendix C, we will show that this can be ‘fixed’, with no change to our final results. Note also that our orderings  and  are different from the usual guiding centre regime, for which  ,  and  .

2 If  tended to a non-zero constant at  , there would be sinusoidal terms here that can just be absorbed by the homogeneous solution for  .

3 As is common in the turbulence literature, we will interchangeably refer to ‘wavenumber’  and ‘scale’  ; the energy change due to a coherent structure at scale  is dominated by the response from  .

4 Note that due to the smallness of  implied by our use of the KREHM equations, there is no contribution to  from parallel magnetic field fluctuations  as appears in the stochastic heating theory of Cerri *et al*. (2021).

5 This assumption is in fact implicit in (4.1)–(4.5) earlier in this section; the minor ions have sufficiently low abundances that they do not affect the KAW dispersion relation nor polarisation properties of the fluctuations.

## Appendix A. Drifts

As well as calculating the behaviour of the velocity as  , we might be interested in the drift velocity of the particle at finite  . It is easy to see that only the terms with  in (3.9) and (3.8) contribute to drifts. Integrating the  term of (3.9) by parts,

(A1)

The first term is the gyroaveraged  drift velocity. Repeating the integration by parts,

(A2)

Comparing this and (3.9), it is easy to see the pattern:

(A3)

a series of corrections to the lowest-order gyroaveraged  velocity (Stephens, Brzozowski & Jenko 2017). Taking  , we obtain the infinite series

(A4)

Differentiating, we obtain a similar series for the drifts in the  direction,

(A5)

with the first term being the gyroaveraged polarisation drift. For  , each successive term in the sum is smaller than the next by a factor  . If  , drifts at all orders are comparable and the series does not converge. As  , the drift part of the motion vanishes since we required  .

## Appendix B. Resonance

We could modify the example in § 3.4 by multiplying by a sinusoid,

(B1)

Expanding the products of cosines and sines appearing in the integral solution for  (3.14), we find that there are terms in the integrands multiplied by  . For  , secular terms therefore appear in the solution for  , scaling with the time interval over which the sinusoidally varying fields are applied. If  , then  in this resonant case. However, in practice, this type of fluctuation clearly involves an overall rate of change of the fields  , so that  and in terms of scalings, the possibility of this resonant type of fluctuation makes little difference to the end result. In the rest of the paper, we ignore this subtlety and assume that our fluctuation is not resonant.

## Appendix C. The case with

Examining our expressions for  and  (see (3.10)–(3.11)), it is possible to see that in fact, the coefficient with  resulting from the second line does not vanish. As mentioned in the main paper, this means that if the fluctuating field is applied over a time longer than  , our ordering in  breaks down, since this secular term will become large. This is not a true resonance, however, and results from the fact that our expansion procedure does not take into account the nearly periodic nature of the system.

To illustrate the issue, we first consider a simplified example. Suppose that the electric field is given by  , constant in time, but depending on  . Then, our equation for  is

(C1)

This has the exact solution  , i.e. the variation of the electric field in space has introduced a frequency shift (Stephens *et al*. 2017). Now, suppose we instead attempted to expand in  : we would instead get  and, at first order,

(C2)

which is secular. To avoid this, we can use the Poincaré–Lindstedt method (Bender & Orszag 2013), introducing a stretched time variable  , with

(C3)

Now, at first order, we have

(C4)

(C5)

where we have, in an abuse of notation, redefined the overdot to mean differentiation by  . The solution that avoids a secular term is  , which agrees with the exact solution up to first order in  .

Our problem unfortunately does not have an readily available exact solution, but we can still apply the Poincaré–Lindstedt method. We order  . Because of the time dependence of the electromagnetic fields, the stretching of time variable  now itself depends slowly on time, with

(C6)

Our differential equation for  (replacing (3.3)) becomes

(C7)

This may be solved (as before) by Fourier transforming in time and back again, and the solution for the velocity is

(C8)

Following the same procedure as in § 3, (see (3.6)–(3.10)) (Fourier transforming in  according to (2.5), applying the Bessel function identity (3.7), and expanding the cosine and combining the resulting sinusoidal terms in the integrands), we obtain

(C9)

A secular term would result if there were a non-zero term in the  integrand of (C9) with no sinusoidal variation – as discussed previously, such a term would break the ordering of our solution if the electric field  remains ‘on’ for a time of order  . In the second integral of (C9), one such term results from the  term, while there is another in the  term. To ensure they cancel, we choose

(C10)

where the second equality follows since the electric field is real; thus,  is real. Because  for  , if  varies only on large scales,

(C11)

This agrees with our previous simplified example with  . In other words, if the electric field varies only a small amount over the gyroradius of the particle,  will also be small (by a factor  ).

With this analysis, it is now proven that even when  or smaller,  (and  , by identical arguments) are exponentially small as  , as is  .
